# Mode-localized accelerometer in the nonlinear Duffing regime with 75 ng bias instability and 95 ng/√Hz noise floor

**DOI:** 10.1038/s41378-021-00340-4

**Published:** 2022-02-07

**Authors:** Hemin Zhang, Milind Pandit, Guillermo Sobreviela, Madan Parajuli, Dongyang Chen, Jiangkun Sun, Chun Zhao, Ashwin A. Seshia

**Affiliations:** 1 The Nanoscience Centre, University of Cambridge, Cambridge, CB3 0FF UK; 2 Silicon Microgravity Ltd., Cambridge Innovation Park, Cambridge, CB25 9PB UK; 3 MOE Key Laboratory of Fundamental Physical Quantities Measurement and Hubei Key Laboratory of Gravitation and Quantum Physics, PGMF and School of Physics, Huazhong University of Science and Technology, 430074 Wuhan, China

**Keywords:** Physics, Electrical and electronic engineering

## Abstract

Mode-localized sensors have attracted attention because of their high parametric sensitivity and first-order common-mode rejection to temperature drift. The high-fidelity detection of resonator amplitude is critical to determining the resolution of mode-localized sensors where the measured amplitude ratio in a system of coupled resonators represents the output metric. Operation at specific bifurcation points in a nonlinear regime can potentially improve the amplitude bias stability; however, the amplitude ratio scale factor to the input measurand in a nonlinear regime has not been fully investigated. This paper theoretically and experimentally elucidates the operation of mode-localized sensors with respect to stiffness perturbations (or an external acceleration field) in a nonlinear Duffing regime. The operation of a mode-localized accelerometer is optimized with the benefit of the insights gained from theoretical analysis with operation in the nonlinear regime close to the top critical bifurcation point. The phase portraits of the amplitudes of the two resonators under different drive forces are recorded to support the experimentally observed improvements for velocity random walk. Employing temperature control to suppress the phase and amplitude variations induced by the temperature drift, 1*/f* noise at the operation frequency is significantly reduced. A prototype accelerometer device demonstrates a noise floor of 95 ng/√Hz and a bias instability of 75 ng, establishing a new benchmark for accelerometers employing vibration mode localization as a sensing paradigm. A mode-localized accelerometer is first employed to record microseismic noise in a university laboratory environment.

## Introduction

Coupled microelectromechanical resonators have been extensively researched^1–4^ and integrated into devices for various engineering applications^5,6^. In the sensing field, a technical approach to high parametric sensitivity has been realized by employing the principle of vibration mode localization in coupled resonators^7–13^. Principally, for mode-localized sensors based on two weakly coupled resonators with structurally symmetric parameters, a symmetry-breaking perturbation introduced to one of the coupled subcomponents will result in drastic shifts in the mode shape, which can be monitored by measuring the amplitude ratio (AR) of the resonators. Thus, the external measurand that induces the perturbation can be sensed. In contrast with the conventional resonant sensing method of monitoring the frequency readout, the AR readout method has been experimentally shown to provide improvement in parametric sensitivity by over two orders of magnitude in comparison^7,11^. Furthermore, mode-localized sensors have exhibited benefits with respect to intrinsic first-order immunity to environmental temperature drift^14,15^. Based on this promising transduction approach, various mode-localized microelectromechanical system (MEMS) sensors have been developed, such as mass sensors^7^, electrometers^10^, accelerometers^15–19^, and magnetic field sensors^20^. Mode-localized accelerometers have demonstrated applicability for seismic monitoring^13^. Further improvements in the noise floor and stability of mode-localized accelerometers are expected to enable further applications, including MEMS gravimetry^21,22^.

Optimization of the amplitude noise floor and stability are critical for the resolution of mode-localized sensors. The sensor output metric can be defined by a motional amplitude ratio for the two resonators. Therefore, in contrast to frequency-modulated sensors, the amplitude signal-to-noise ratio (SNR) of each resonator is of more specific interest for mode-localized sensors. It is known that frequency and amplitude stabilities are highly sensitive to the oscillation amplitude^23–25^, and an increased actuation force can improve the SNR, whereas there is a high possibility that the resonator is driven into a nonlinear Duffing regime. In the nonlinear regime, the frequency response is reshaped by conservative or dissipative nonlinearities, and the vibration amplitude of the resonator at a specific frequency is determined by the previous state, resulting in nonidentical forward and backward frequency responses^26^. Consequently, the amplitude-to-frequency effect is evident in the nonlinear regime. The so-called bifurcation points (top or bottom bifurcation points) where the frequency (*f*) and phase (*φ*) fulfill the criterion of *∂f/∂φ* = 0^27^ result in reduced sensitivity to phase noise. Consequently, the bias instability can be improved, which has been experimentally demonstrated^28,29^. Experimental studies have indicated that the input-referred AR noise floor can be improved by operating in this regime to a certain extent^30^.

A theoretical study on the nonlinear sensitivities of the amplitude ratio of the coupled resonators was previously shown^31^ for the special condition of AR~1, i.e., around the veering point. However, the AR sensitivity^13,32^ with respect to the stiffness perturbation and its resolution^33^ vary with changes in amplitude ratio (or stiffness perturbation). Further analysis is thus necessary to provide expressions applicable for operation in linear and nonlinear regimes. Moreover, the assumptions in ref. ^31^ did not support the scenario of ultraweak coupling (cases where the quality factor is not high enough to be ignored), which is specifically needed for high-resolution mode-localized sensors^33,34^, and further work, including experimental verification, is necessary to probe the conclusion that the resolution will be improved by continuously increasing the vibration amplitude.

In this paper, we discuss the AR scale factor for large vibration amplitudes in linear and nonlinear Duffing regimes. A general expression of the backbone nature (frequency-amplitude effect) and the AR scale factor of the coupled resonators suitable for both linear and nonlinear cases are derived and experimentally demonstrated. The AR scale factor remains relatively constant in the linear regime, whereas it drops considerably when the resonator enters the nonlinear regime. The optimal operation amplitude is shown to be close to the critical amplitude, where the sensor demonstrates the best noise floor and stability. A further increase in the drive amplitude in the nonlinear regime will not result in continuous improvement of the noise floor and stability. This insight enables optimization of device performance with a prototype demonstrating a noise floor of 95 ng/√Hz and a bias instability of 75 ng, allowing for the first recording of a low-frequency microseismic background using a mode-localized MEMS accelerometer.

## Results and discussion

### Device description

**Fig. 1:**
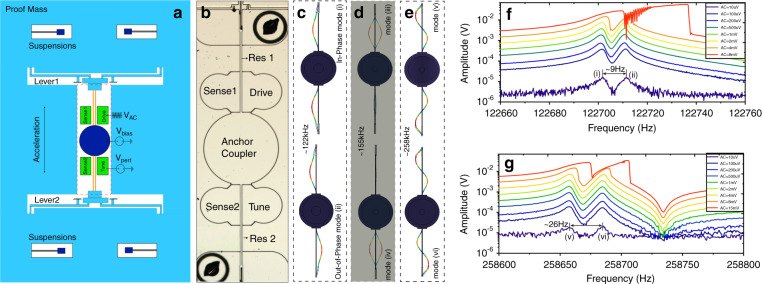
Device description and characterization. **a** Schematic of the mode-localized accelerometer. **b** Optical image of the coupled resonators. **c**–**e** The simulated vibration modes of the two resonators. Modes in **c** and **e** are of interests in this paper. **f** Frequency responses of Res 1 (the primary resonator that is directly driven) in the lower-order and **g** the higher-order modes of interests with different drive AC signals, with the initial AR value in the linear regime close to the veering point (AR~1)

The mechanical element of the mode-localized accelerometer consists of two spring-supported masses and two structurally symmetric double-ended-tuning-fork (DETF) resonators fixed to the same center anchor, as shown in Fig. 1a. The proof mass is mechanically connected to one end of the DETF resonator through a lever mechanism for inertial force amplification. An optical image of the coupled resonators is shown in Fig. 1b, and the fabrication cross-section can be found in ref. ^21^. Dimensions of the device are provided in Supplementary Table I. For the lower-order vibration modes (i) and (ii) in Fig. 1c and the higher-order modes (v) and (vi) in Fig. 1e, the two DETF tines move in a parallel direction, and the coupling forces arising from the DETF vibrations are transmitted to the anchor as shear forces, resulting in small in-plane deformations of the coupler, which is three orders of magnitude smaller than that of the tines. Energy transfer (mode coupling) between Res 1 and Res 2 is thus realized through coupler in-plane deformation^35^. In contrast, when the two tines move in opposite directions, the shear force at the end of the tines is largely reduced so that the two resonators cannot be effectively coupled together, as shown by modes (iii) and (iv) in Fig. 1d, in which only one resonator is in obvious vibration so that these two modes are not of interest. The degree of modal coupling (coupling factor) can be set practically by adjusting the diameter of the anchor coupler. The resonators are capacitively actuated and sensed through the electrodes on either side of the DETF tines. A 10 V DC voltage is applied to the central anchor as the bias voltage. The motional current is translated to a voltage via trans-impedance amplifiers. A variable voltage is applied to the tuning electrode integrated with Res 2 to provide electrostatic negative stiffness perturbations to Res 2 so that the AR operation point can be manually adjusted. The test setup can be found in Supplementary Material Fig. S1.

The amplitude-frequency responses of Res 1 (the primary resonator that is directly driven) in the lower-order and higher-order modes of interest with different drive AC amplitudes are shown in Fig. 1f, g, respectively. Modes (i) and (ii) indicate linear resonant frequencies of *f*_1_ ≈ 122,703 Hz and *f*_2_ ≈ 122712 Hz. A frequency split of ~9 Hz is observed, demonstrating a coupling factor^36^ of *κ* ≈ (*f*_2_ – *f*_1_)⧸*f*_1_ = 7.4 × 10^−5^) and a *Q*κ value of ~3.0, as the Q factor is estimated to be 39 k. For the higher-order modes, the coupling factor is 10.3 × 10^−5^. With the increase in the drive AC signal, the response magnitudes will increase and enter a nonlinear Duffing regime if the AC signal is higher than a critical value. The responses of Res 2 can be found in Supplementary Material Fig. S2. To explore the optimized operation amplitude of the coupled resonators, the AR scale factor expression with respect to the stiffness perturbation will be theoretically derived in the following section.

### Theoretical model

For a two-degree-of-freedom weakly coupled resonating system, it is normally assumed that the two resonators have identical initial structural parameters, i.e., identical mass: *m*_1_ = *m*_2_ = *m*:, stiffness: *k*_1_ = *k*_2_ = *k* and damping: *c*_1_ = *c*_2_ = *c* in a lumped mechanical model^36,37^, and only one resonator is driven by the harmonic force. The dissipative nonlinearity and the cubic nonlinearity of the coupler can be ignored because no significant amplitude-dependent damping is observed for our device, and the deformation of the coupler is very small. The nonlinear springs for the two resonators are defined as $k_1 = k\left( {1 + \gamma x_1^2} \right)$, and $k_2= k\left( {1 + \gamma x_2^2} \right)$, where *γ* is the cubic nonlinear coefficient, which is assumed to be identical for the two resonators. The nonlinear dynamic equations of the coupled resonators are given by:

1a $$\ddot x_1 + \frac{{\omega _0}}{Q}\ddot x_1 + \omega _0^2\left( {1 + \kappa + \gamma x_1^2} \right)x_1 - \kappa \omega _0^2x_2 = f/m$$

1b $$\ddot x_2 + \frac{{\omega _0}}{Q}\ddot x_2 + \omega _0^2\left( {1 + \kappa + \delta + \gamma x_2^2} \right)x_2 - \kappa \omega _0^2x_1 = 0$$

where *x*_1_and *x*_2_ are the displacements of the two resonators, $\omega _0 = \sqrt {k/m}$ is the initial natural frequency, *δ* = ∆*k/k is* the stiffness perturbation to Res 2, $Q = m\omega _0/c$ is the quality factor, and $f = F{\mathrm{sin}}\left( {\omega _dt + \theta } \right)$ is the force applied to Res 1. The displacements are written as:

2a $$x_1 = X_1(t){\mathrm{sin}}(\omega t)$$

2b $$x_2 = X_2(t){\mathrm{sin}}(\omega t + \Phi (t))$$

where *X*_1_(*t*), *X*_2_(*t*), and Φ(*t*) are slow variables with time scales much slower than 2*π*/*ω*. According to (2a) and (2b), we have the following expressions:

3a $$\dot x_1 = \omega X_1{\mathrm{cos}}\left( {\omega t} \right) + \dot X_1{\mathrm{sin}}(\omega t)$$

3b $$\ddot x_1 = - \omega ^2X_1{\mathrm{sin}}\left( {\omega t} \right) + 2\omega \dot X_1{\mathrm{cos}}\left( {\omega t} \right) + \ddot X_1{\mathrm{sin}}\left( {\omega t} \right)$$

3c $$x_1^3 = X_1^3{\mathrm{sin}}^3\left( {\omega t} \right) = \frac{{X_1^3}}{4}\left[ {3{\mathrm{sin}}\left( {\omega t} \right) - {\mathrm{sin}}\left( {3\omega t} \right)} \right]$$

4a $$\dot x_2 = \left( {\omega + \dot \Phi } \right)X_2{\mathrm{cos}}\left( {\omega t + \Phi } \right) + \dot X_2{\mathrm{sin}}(\omega t + \Phi )$$

4b $$\begin{array}{ll}\ddot x_2 = -\, \left( {\omega + \dot \Phi } \right)^2X_2{\mathrm{sin}}\left( {\omega t + \Phi } \right) + \,2\left( {\omega + \dot \Phi } \right)\dot X_2{\mathrm{cos}}\left( {\omega t + \Phi } \right) \\+ \,\ddot X_2{\mathrm{sin}}(\omega t + \Phi )\end{array}$$

4c $$\begin{array}{ll}x_2^3 = X_2^3{\mathrm{sin}}^3\left( {\omega t + \Phi (t)} \right) \\= \frac{{X_2^3}}{4}\left[ {3{\mathrm{sin}}\left( {\omega t + \Phi (t)} \right) -\, {\mathrm{sin}}\left( {3\left( {\omega t + \Phi (t)} \right)} \right)} \right]\end{array}$$

By substituting (3a) to (4c) into the dynamic Eqs. (1a) and (1b), ignoring the higher-order term (3*ωt*) and using the harmonic balance principle^8,31^, the following equations are trivially obtained:

5a $$\begin{array}{ll}\ddot X_1 +\, \frac{{\omega _0}}{Q}\dot X_1 + X_1\left[ { - \omega ^2 + \left( {1 + \kappa } \right)\omega _0^2 + \frac{3}{4}\omega _0^2\gamma X_1^2} \right] \\-\, \kappa \omega _0^2X_2{\mathrm{cos}}{\it{\Phi }} = \frac{F}{m}{\mathrm{cos}}\theta\end{array}$$

5b $$2\omega \dot X_1 + \frac{{\omega _0}}{Q}\omega X_1 - \kappa \omega _0^2X_2{\mathrm{sin}}{\it{\Phi }} = \frac{F}{m}{\mathrm{sin}}\theta$$

5c $$\ddot X_2 + \frac{{\omega _0}}{Q}\dot X_2 + X_2\left[ { - \left( {\omega + {{\dot \Phi }}} \right)^2 + \, \omega _0^2 \left( {1 + \kappa + \delta } \right)} \right] - \,\kappa \omega _0^2X_1{\mathrm{cos}}{{\Phi }} + \frac{3}{4}\gamma \omega _0^2X_2^3 = 0$$

5d $$2\left( {\omega + {{\dot \Phi }}} \right)\dot X_2 + \frac{{\omega _0}}{Q}\omega X_2 + \kappa \omega _0^2X_1{\mathrm{sin}}{{\Phi }} = 0$$

The first-order ($\dot X_1$, $\dot X_2$) and second-order ($\ddot X_1$, $\ddot X_2$) differential terms have little influence on the steady-status amplitudes and thus can be ignored. According to (5b) and (5d) and by ignoring ${{\dot \Phi }}$ in the steady-state (*X*_1_, *X*_2_ and ${{\dot \Phi }}$ changes very slowly compared to the resonant frequency), the phase difference is given by:

6a $${\mathrm{sin}}{\it{\Phi }} = \pm \frac{\omega }{{\kappa Q\omega _0}}\frac{1}{{{\mathrm{AR}}}} \approx \pm \frac{1}{{{\mathrm{AR}}}} \cdot \frac{1}{{\kappa Q}}$$

6b $${\mathrm{cos}}{\it{\Phi }} \approx \pm \left( {1 - \frac{1}{2}\left( {\frac{1}{{{\mathrm{AR}}}} \cdot \frac{1}{{\kappa Q}}} \right)^2} \right)$$

where AR is defined as *X*_1_/*X*_2_. The phase difference between resonators is inversely proportional to the amplitude ratio and the value of *κQ*. Normally, Φ = 0 indicates the in-phase mode and ${\it{\Phi }} = \pi$ indicates the out-of-phase mode. The weakly coupled resonators operate in the exact in-phase mode or out-of-phase mode only in the case of AR >> 1. Otherwise, the coupled resonators will demonstrate specific phase delays compared to the standard in-phase or out-of-phase mode. Equations (5a) and (5c) can be rearranged as:

7a $$- \frac{{\omega ^2}}{{\omega _0^2}} + \left( {1 + \kappa } \right) + \frac{{3\gamma X_1^2}}{4} - \frac{{\kappa X_2{\mathrm{cos}}{\it{\Phi }}}}{{X_1}} = \frac{{F{\mathrm{cos}}\theta }}{{\omega _0^2mX_1}}$$

7b $$- \frac{{\omega ^2}}{{\omega _0^2}} + \left( {1 + \kappa + \delta } \right) + \frac{{3\gamma X_2^2}}{4} - \frac{{\kappa X_1{\mathrm{cos}}{\it{\Phi }}}}{{X_2}} = 0$$

According to (7a) and (7b), giving *θ* a value of 90° so that the resonator vibrates at the top bifurcation point of the 1st mode^30^, a comprehensive expression between the resonant frequency (*ω*), stiffness perturbation (*δ*), amplitude (*X*_1_), and amplitude ratio (AR = *X*_1_/*X*_2_) is obtained:

8 $$\begin{array}{ll}\frac{{\omega ^2}}{{\omega _0^2}} = \left( {1 + \kappa + \frac{\delta }{2}} \right) + \frac{{3\gamma X_1^2}}{8}\left( {1 + 1/{\mathrm{AR}}^2} \right)\\ \mp\, \frac{\kappa }{2}\left( {1 - \frac{1}{2}\left( {\frac{1}{{{\mathrm{AR}}}} \cdot \frac{1}{{\kappa Q}}} \right)^2} \right)\left( {\frac{1}{{{\mathrm{AR}}}} + {\mathrm{AR}}} \right)\end{array}$$

Based on (8), the backbone nature (frequency-amplitude response) of the nonlinear resonance is also dependent on the amplitude ratio. Furthermore, the AR expression can be derived from (7a) and (8):

9 $$\left( {{\mathrm{AR}} - \frac{1}{{{\mathrm{AR}}}}} \right)\left( {1 - \frac{1}{2}\left( {\frac{1}{{{\mathrm{AR}}}} \cdot \frac{1}{{\kappa Q}}} \right)^2} \right) = {\mathrm{AR}} - \frac{1}{{{\mathrm{AR}}}} - \frac{1}{{2\left( {\kappa Q} \right)^2{\mathrm{AR}}}} + \frac{1}{{2\left( {\kappa Q} \right)^2{\mathrm{AR}}^3}}$$

The generalized AR scale factor to the stiffness perturbation (*δ*) in both the linear and nonlinear regimes is thus given by:

10 $$\frac{{\partial {\mathrm{AR}}}}{{\partial \delta }} = \frac{1}{{\kappa \left( {1 + \frac{{\left( {1 + \frac{1}{{2\left( {\kappa Q} \right)^2}}} \right)}}{{{\mathrm{AR}}^2}} - \frac{3}{{2\left( {\kappa Q} \right)^2{\mathrm{AR}}^4}}} \right) + \frac{{3\gamma X_1^2}}{{2{\mathrm{AR}}^3}}}}$$

In some special cases where *κQ* ≫ 1, which is normal for researchers pursuing mode-localized sensors, the term $\frac{1}{2}\left( {\frac{1}{{{\mathrm{AR}}}} \cdot \frac{1}{{\kappa Q}}} \right)^2$ in (9) is negligible, i.e., Φ ≈ 0; thus, the AR sensitivity with respect to *δ* is simplified as:

11 $$\left. {\frac{{\partial {\mathrm{AR}}}}{{\partial \delta }}} \right|_{{\mathrm{nonlinear}}} = \frac{1}{{\kappa \left( {1 + \frac{1}{{{\mathrm{AR}}^2}}} \right) + \frac{{3\gamma X_1^2}}{{2{\mathrm{AR}}^3}}}}$$

Expression (11) provides a simplified expression for AR sensitivity to stiffness perturbation. As our device shows a value of *κQ*~3, there would be a slight estimation error between the simplified expressions (11) and (10). However, the estimation error is small and is on the order of <3% if AR > 2, which is an acceptable approximation. Therefore, the simplified expression (11) is considered in the following numerical analysis. In a linear regime, the cubic nonlinear parameter can be set as *γ* = 0, which trivially leads to an expression of the AR sensitivity in the linear regime:

12 $$\left. {\frac{{\partial {\mathrm{AR}}}}{{\partial \delta }}} \right|_{{\mathrm{linear}}} = \frac{1}{{\kappa \left( {1 + \frac{1}{{{\mathrm{AR}}^2}}} \right)}}$$

which is in good alignment with our previous theoretical work on mode-localized sensors operating in a linear regime^33^.

**Fig. 2:**
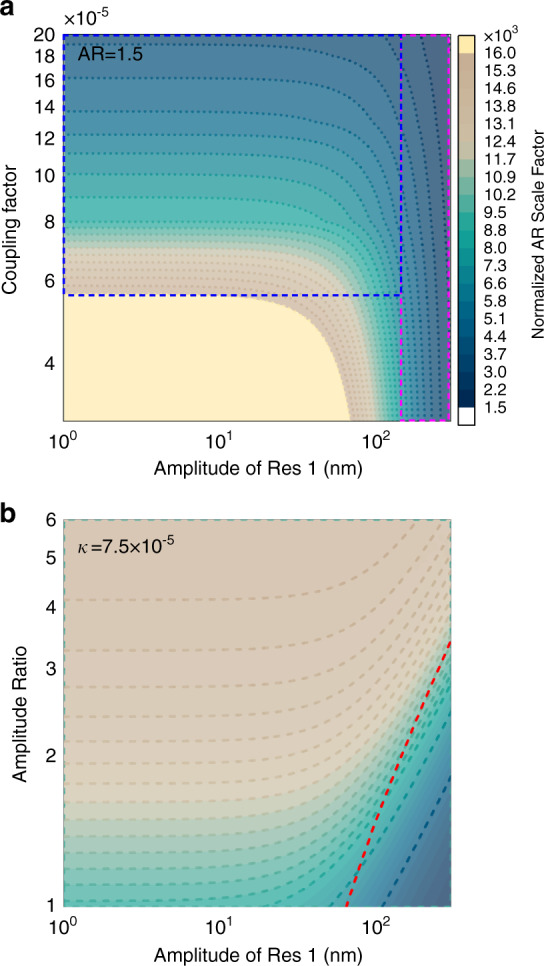
Simulated amplitude ratio scale factor. **a** Contour plots of the numerically simulated AR scale factor versus the amplitude of Res 1 and coupling factor. **b** Contour plots of the numerically simulated AR scale factor versus the amplitude of Res 1 and AR. The dashed blue block in **a** depicts the zone where the coupling factor dominates the scale factor and the dashed violet block depicts the zone where the driven amplitude dominates the response. The Q factor is set as 40k, and the cubic nonlinear parameter is γ = 10^10^m^−2^. The red dashed line in **b** indicates the amplitude threshold that the AR scale factor decreases to 95% of the initial value in the initial linear regime

According to (10), the AR scale factor with respect to the stiffness perturbation in a nonlinear regime is determined by the coupling factor *κ*, the vibration amplitude of Res 1 (*X*_1_), and the amplitude ratio. The contour plot of the AR scale factor with different *X*_1_ and *κ* values can be seen in Fig. 2a. The AR operation regime can be separated into two regimes, i.e., a coupling factor dictated zone (the blue dashed block) and an amplitude-determined zone (the violet dashed block). In the coupling factor dictated zone, it is evident that a lower coupling factor results in a higher scale factor. However, in the amplitude-determined zone, the AR scale factors are similar to various *κ* values. This indicates that too large a vibration amplitude in the nonlinear regime could result in a reduced performance for the scale factor of mode-localized sensors.

Once the coupling factor is confirmed, which is the normal case for mechanically coupled resonators^10–13^, the AR scale factor will only be defined by the vibration amplitude of the driven resonator (*X*_1_) and the amplitude ratio. The contour plot in Fig. 2b shows that there is a higher AR scale factor with a larger amplitude ratio, in good correspondence with previously reported cases in the linear regime^13^. Again, the scale factor drops considerably when the vibration amplitude is higher than a threshold value. If we set decreasing to 95% of the initial AR scale factor as the threshold, this vibration amplitude can be derived based on (11):

13 $$X^2_{{\mathrm{threshold}}} = \frac{{2(\sqrt 2 - 1)\left( {{\mathrm{AR}}^3 + {\mathrm{AR}}} \right)\kappa }}{{3\gamma }}$$

### Nonlinear characterization in an open-loop configuration

**Fig. 3:**
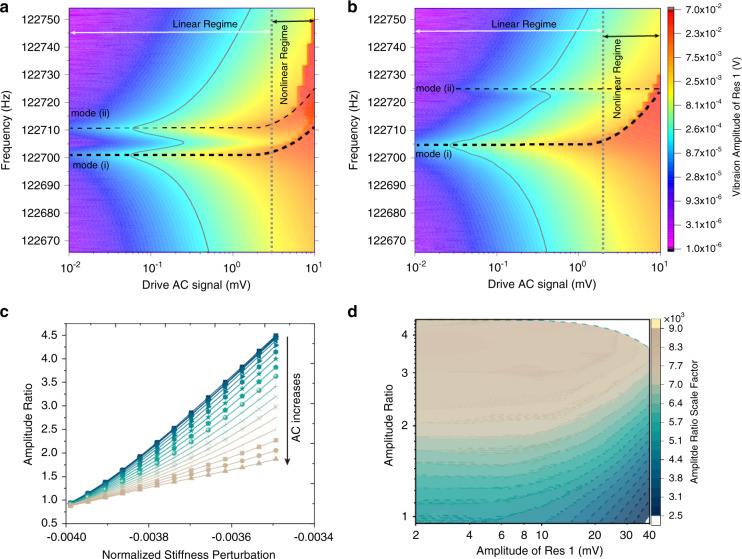
Open-loop and closed-loop nonlinear characterization. **a** Experimental measurement of the sweep-up amplitude-frequency responses of Res 1 with different drive AC values, with an initial condition of AR = 1.1 and AR = 4.2 (**b**). The dotted curves in **a** and **b** represent the backbone nature. **c** Amplitude ratio as a function of the normalized stiffness perturbation with different drive AC voltages. **d** The contour plot of the AR scale factor versus amplitude of Res 1 and the amplitude ratio

The detailed amplitude-frequency responses (sweep-up) of Res 1 in the lower-order vibration modes (i) and (ii) with variable drive AC signals at different initial AR values were recorded (Fig. 3a, b) to investigate the critical amplitude and backbone nature. The linear and nonlinear regimes can be clearly differentiated. The backbone curve is shown by the dashed line. In Fig. 3a, the initial amplitude ratio is 1.1, and Res 1 goes into the nonlinear regime when the drive AC is higher than 3 mV. When the initial amplitude ratio is much higher than 1, for instance, AR = 4.2, which can be obtained by tuning the voltage applied to Res 2, as shown in Fig. 3b, it can be seen that the resonator goes into the nonlinear regime when the drive AC is higher than 2 mV (not 3 mV as when AR = 1.1 in Fig. 3a). Note that the vibration energy of the coupled resonators is more likely confined in Res 1 when the system is asymmetric (AR >>1). Correspondingly, both mode (i) and mode (ii) in Fig. 3a illustrate backbone characteristics, while only mode (i) in Fig. 3b demonstrates similar amplitude-to-frequency dependence. It can also be derived by (8) that the nonlinear shift in resonant frequency is influenced by the AR value. The contour plots of the frequency responses of Res 2 are provided in Supplementary Fig. S3.

### Nonlinear scale factor characterization in closed-loop configuration

To verify the proposed theory on AR sensitivity, we manually tune the perturbation voltage applied to Res 2 in a closed-loop configuration to obtain electrostatic stiffness perturbations while the sensor is mounted horizontally, i.e., without any external acceleration input. The closed-loop configuration is realized by controlling Res 1 to vibrate at a specific phase through the phase-lock-loop function integrated as part of the Zurich MFLI lock-in amplifier (see Supplementary Fig. S1 for more details). The vibration amplitudes of the two resonators at resonance are demodulated and recorded. The amplitude ratio is then calculated based on the collected amplitudes. To obtain different amplitude ratios, the voltage (**V**_**pert**_) applied to Res 2 is tuned from −4.7V to −5.7 V. The net stiffness perturbation is ${{{\mathbf{k}}}}_{{{{\mathbf{pert}}}}} = - {{{\mathbf{\varepsilon }}}}_0{{{\mathbf{A}}}}\left( {{{{\mathbf{V}}}}_{{{{\mathbf{pert}}}}} - {{{\mathbf{V}}}}_{{{{\mathbf{bias}}}}}} \right)^2/{{{\mathbf{g}}}}^3$, and considering the initial softening effect from the bias voltage ${{{\mathbf{k}}}}_{{{{\mathbf{neg}}}}} = - {{{\mathbf{\varepsilon }}}}_0{{{\mathbf{A}}}}\left( {{{{\mathbf{V}}}}_{{{{\mathbf{bias}}}}}} \right)^2/{{{\mathbf{g}}}}^3$, the normalized stiffness perturbation is ${{{\mathbf{\delta }}}} = - {{{\mathbf{\varepsilon }}}}_0{{{\mathbf{AV}}}}_{{{{\mathbf{pert}}}}}\left( {{{{\mathbf{V}}}}_{{{{\mathbf{pert}}}}} + 2{{{\mathbf{V}}}}_{{{{\mathbf{bias}}}}}} \right)/{{{\mathbf{g}}}}^3/{{{\mathbf{k}}}}$, where **k** is the stiffness of the DETF resonator, **ε**_**0**_ is the permittivity, **A** is the electrode area, and **g** is the gap between coupling electrodes. As expected, an increased drive AC signal results in a lower AR scale factor, which can be read from the curve slopes in Fig. 3c. The AR scale factor for the stiffness perturbation is calculated using the gradients of the AR fitting curves in Fig. 3c. The contour plot of the AR scale factor versus the amplitude ratio with different drive AC values is shown in Fig. 3d, which perfectly reproduces the theoretical prediction in Fig. 2b.

### The mode-localized accelerometer in the lower-order mode

Previous theories and experiments have provided clear evidence that continuously increasing the vibration amplitude to move the resonator into the nonlinear regime will result in AR scale factor reduction. However, there are plenty of theoretical and experimental results showing improvements in the amplitude and frequency noise floor with a large drive amplitude in the nonlinear regime^28,38,39^. In this section, we will explore the input-referred AR noise floor and stability of the mode-localized accelerometer in low- and large-amplitude operation regimes to determine the optimal operation amplitude for mode-localized sensors.

To test the AR sensitivity with respect to the external acceleration, the device is placed on a high-precision tilting platform^40^ so that there is a component of gravitational acceleration acting along the sensitive axis of the accelerometer resulting in differential stiffness perturbations to Res 1 and Res 2. The chip and the front-end electronics are housed in a shielded box to prevent external electromagnetic interference for the stability measurement. The experiments are operated under two conditions: (a) at room temperature without any temperature control and (b) with temperature control. Although mode-localized sensors have demonstrated the property of common-mode rejection to the temperature drift, temperature control employed here is to control the stability of the electronics, such as the feedback resistors of the trans-impedance amplifiers. Furthermore, as a phase-locked loop is selected for realizing the closed-loop configuration, the phase-locking precision is important for the final resolution of the mode-localized sensors, which performs better in a thermally stable environment. Two-level temperature control is implemented, i.e., a chamber level that is used to control the temperature of the surrounding environments in the shield within ±1 mK with a setpoint of 35 °C and a chip level to regulate the chip/board temperature to ±5 mK with a setpoint of 45 °C.

**Fig. 4:**
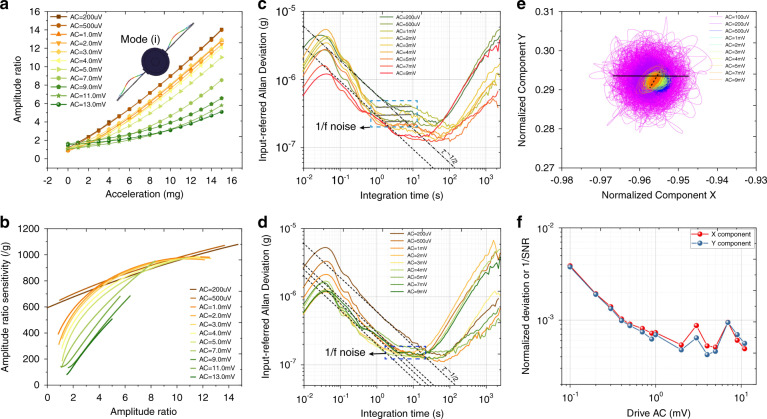
Performance of the mode-localized accelerometer in the lower-order mode (i). **a** Amplitude ratio as a function of the external acceleration with different drive AC voltages. **b** AR scale factor versus amplitude ratio with different drive AC voltages. Input-referred Allan deviation with various drive AC voltages in the condition of no temperature control (**c**) and chamber-level temperature control (**d**). The dashed lines in **d** represent the thermal noise floor. **e** The portrait of the quadratures of the output voltage of Res 1. **f** The normalized deviation of the X and Y components versus varying AC values

The measured AR values in the lower-order mode (i) as a function of the external acceleration under different drive AC voltages are shown in Fig. 4a, and the AR scale factors are shown in Fig. 4b. The amplitudes of the two resonators were collected for 2 h with a sampling rate of 209.3 Hz. The input-referred Allan deviation of the accelerometer in the lower-order mode (i) under the conditions of no temperature control and chamber-level temperature control can be found in Fig. 4c, d. Each curve is obtained with a fixed acceleration input, a constant voltage perturbation to Res 2, and varying drive AC amplitudes when the AR value is ~1.5. The dotted lines in the Allan deviation curves with a slope of τ^−1/2^ indicate the net thermal noise^38,41^, which involves thermomechanical noise and readout electronic noise.

The results under the condition of resonators working at room temperature in the lower-order mode (i), shown in Fig. 4c, suggest that white noise can be suppressed by enlarging the drive amplitude. The results show significant 1*/f* noise for integration times between 1 and 10 s, setting a limit to the measured bias instability. Increasing the drive amplitude results in an improvement in the bias instability, and this behavior is consistent with previous observations in MEMS gyroscopes^42^. The bias instability is improved from 250 ng with a drive level of 200 μV to 130 ng with a drive level of 9 mV.

The results under the condition of the resonators working in the lower-order mode (i) with chamber-level temperature control, shown in Fig. 4d, again indicate the effectiveness of enlarging the drive amplitude to suppress the detection noise. There will be no further improvement on the noise floor when the drive amplitude is higher than 3 mV, which is the drive amplitude that results in the critical vibration amplitude according to Fig. 3a. Although 1*/f* noise still appears within the integration time between 1 and 10 s, it is much lower than that in the condition of no temperature control, and further enlarging the drive amplitude does little to decrease the 1*/f* noise. Furthermore, it can be seen that the lower bound limit of the Allan deviation cannot be much improved by increasing the drive amplitude.

The two quadratures (V_*x*_, V_*y*_) of the voltage output of Res 1 were extracted using a lock-in amplifier. The normalized X and Y components with varying drive amplitudes are calculated with the formulae ${{{\mathbf{V}}}}_{{{\boldsymbol{x}}}}/\sqrt {{{{\mathbf{V}}}}_{{{\boldsymbol{x}}}}^2 + {{{\mathbf{V}}}}_{{{\boldsymbol{y}}}}^2}$ and ${{{\mathbf{V}}}}_{{{\boldsymbol{y}}}}/\sqrt {{{{\mathbf{V}}}}_{{{\boldsymbol{x}}}}^2 + {{{\mathbf{V}}}}_{{{\boldsymbol{y}}}}^2}$, respectively, and are shown in Fig. 4e. The area of this phase portrait represents the amplitude noise level or SNR of the resonator^38,42^. In good correspondence with what has been observed in the Allan deviation curves, the noise level will decrease (phase portrait area) as the drive amplitude increases. Furthermore, we can see in Fig. 4f that the SNR almost linearly increases when the drive amplitude is lower than 2 mV and demonstrates no obvious further improvement when the drive amplitude is higher than 2 mV, which can be used to explain the noise characteristics in Fig. 4c, d.

### Spectrally portraying the microseismic background in the higher-order mode

**Fig. 5:**
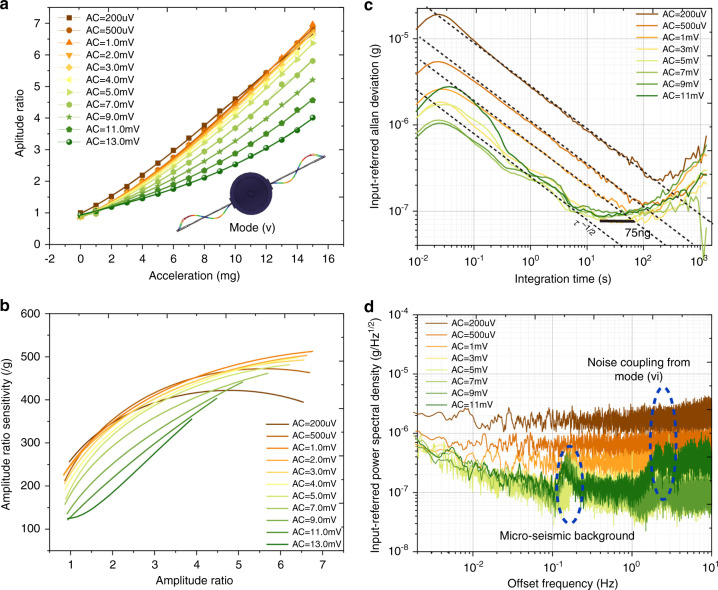
Performance of the mode-localized accelerometer in the higher-order mode (v). **a** Amplitude ratio as a function of the external acceleration with different drive AC voltages. The markers are experimental results, and the solid curves are from the fitting data. **b** AR scale factor versus amplitude ratio with different drive AC voltages. **c** Input-referred Allan deviation and **d** noise power spectral density with various drive AC voltages with board-level and chamber-level temperature control. The dashed lines in **c** represent the thermal noise. The sensitive axis of the sensor is oriented in the horizontal configuration with an AR value of ~1.3–1.6

Compared to the lower-order mode, the higher-order mode shows a lower AR scale factor with respect to the acceleration because of the higher coupling factor^43^, as demonstrated in Fig. 1g. The measured AR scale factors in mode (v) can be found in Fig. 5a, b. Board-level and chamber-level temperature control were employed in this measurement. The input-referred Allan deviation curves under different drive amplitudes are shown in Fig. 5c. A similar effect of improvement on the noise limit when increasing the drive amplitude is observed in this higher-order mode. Benefiting from the implementation of board-level temperature control, 1*/f* noise is further suppressed, and a bias instability of 75 ng is obtained when the drive level is 5 mV. The input-referred noise power spectral densities (NPSD) of the accelerometer in mode (v) with varying drive amplitudes can be found in Fig. 5d. Prominent peaks with a magnitude of ~300 ng√Hz at a frequency of 0.1–0.3 Hz are observed when the drive amplitude is higher than 3 mV, which is attributed to the microseismic background^21,44^ in the university laboratory. This measurement is a good reproduction of our previous report (Fig. S10 in ref. ^21^) using a frequency-modulated accelerometer. The best-collected NPSD is 95 ng√Hz at 1 Hz when the drive amplitude is 5 mV. In the nonlinear regime with a drive amplitude higher than 5 mV, the noise floor will not be further improved or degraded. The peaks arising at frequencies between 20 and 30 Hz are coupled from mode (vi)^30^, which limits the device bandwidth to <10 Hz.

### Linearity error in the nonlinear Duffing regime

**Fig. 6:**
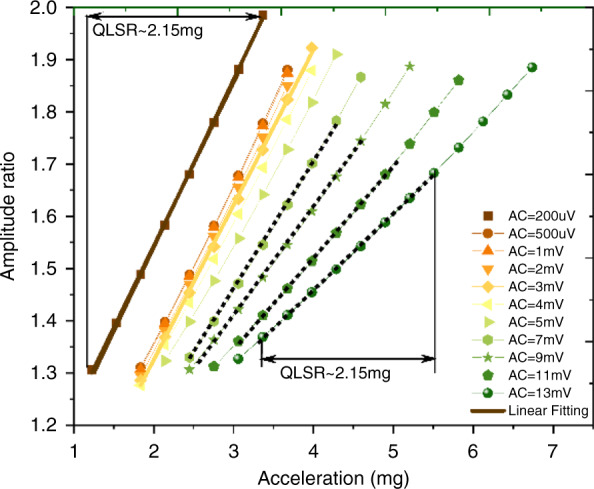
Linear sensing range analysis for the mode-localized accelerometer in the higher-order (v). The term QLSR highlighted in the figure is an abbreviation for quasi-linear sensing range. The marker points in this figure are from the fitting data in Fig. 5a around AR~1.5

Due to the variations in the AR sensitivity, as shown in Figs. 4b and 5b, the AR readout is not constantly a linear function of the external acceleration. There are lower linearity errors when the amplitude ratio is far away from the veering zone where AR~1^45^; however, the resolution and stability will drop in this relatively linear regime (AR >> 1)^33,35^. In this paper, we set the working point at AR ≈ 1.5 to approach the best resolution even associated with the tradeoff of worse linearity. We take the higher-order mode (v) as an example to evaluate the quasi-linear sensing range around the working point, as shown in Fig. 6. The quasi-linear sensing range (with maximum linearity < 1%) around the working point is limited to ~2.15 mg regardless of whether the resonator is operating in the nonlinear Duffing regime.

Although the mode-localized accelerometer reported in this paper demonstrates a limited linear sensing range due to the inherent characteristics, there are several ways to realize an extended dynamic range. For instance, the previous work^13^ has demonstrated an extended linear sensing range using a differential amplitude ratio readout.

## Discussion

This paper investigates the optimal operation amplitude for mode-localized accelerometers. It is found that increasing the drive amplitude can significantly improve the noise floor and velocity random walk if the vibration of the resonator is not higher than the critical Duffing amplitude. In a nonlinear Duffing regime, the AR readout demonstrates a similar performance as that around the critical amplitude regardless of the value of the drive amplitude. By employing two-level temperature control, the 1*/f* noise can be suppressed, and the bias instability is improved by a factor of ~2. While working in the higher-order mode, a bias instability of 75 ng and noise floor of 95 ng/√Hz are obtained. These results are the best-collected metrics for accelerometers employing the mode-localization paradigm to date, and they are also comparable with state-of-the-art frequency-modulated resonant accelerometers^21,46^. Further work on optimizing electronic noise, e.g., the power source noise that contributes to the Lorentzian profile of the resonator via bias and perturbation voltages, can be conducted to approach the ultraprecise level for the application of MEMS gravimeters^47,48^. This mode-localized accelerometer is the first to practically display the microseismic background in the frequency interval 0.1–0.5 Hz with a level of 300 ng/√Hz.

However, as the frequency split between the two modes of interest is very low <50 Hz (~9 Hz for the lower-order mode and ~27 Hz for the higher-order mode) to achieve a lower coupling factor, the noise of the neighboring mode will be coupled to the working mode, resulting in a relatively low working bandwidth and higher short-term Allan deviation. To solve this problem, one potential solution is to increase the frequency split, which means that the natural frequency should be enlarged to maintain a low coupling factor. At the same time, the inertial force-induced stiffness sensitivity should not be significantly decreased, thus introducing a challenge for mechanical design. The other solution is to use the noise of the neighboring mode to calibrate the noise of the working mode^49^. It should be noted that the theory in this paper is limited by the assumptions inherent in the model, and the mode-localization phenomenon is still evident. In addition, there is a possibility to design special resonator structures to lower mechanical nonlinearity so that the operational vibration amplitude can be enlarged and the sensor performance can be consequently improved.

## Supplementary information

Supplemental Material
