# Supplementary material for: Mode-localized accelerometer in the nonlinear Duffing regime with 75 ng bias instability and 95 ng/√Hz noise floor
Source: Microsyst Nanoeng. 2022 Feb 7;8:17. doi: 10.1038/s41378-021-00340-4 (PMC8818770; doi:10.1038/s41378-021-00340-4)
Supplement: Supplementary file 1 — Supplemental Material [file 41378_2021_340_MOESM1_ESM.pdf]

Supplementary Materials

Mode-Localized Accelerometer in the Nonlinear Duffing Regime with 75 ng Bias Instability and 95 ng/√Hz Noise floor

Hemin Zhang[1], *Milind Pandit*[2], *Guillermo Sobreviela*[2], *Madan Parajuli*[1], *Dongyang Chen*[1], *Jiangkun Sun*[1], *Chun Zhao*[3], *and Ashwin A. Seshia*[1*]

[1]The Nanoscience Centre, University of Cambridge, Cambridge CB3 0FF, U.K.
[2]Silicon Microgravity Ltd., Cambridge Innovation Park, Cambridge CB25 9PB, U.K.
[3]MOE Key Laboratory of Fundamental Physical Quantities Measurement and Hubei Key Laboratory of Gravitation and Quantum Physics, PGMF and School of Physics, Huazhong University of Science and Technology, Wuhan 430074, China.
Correspondence: Ashwin A. Seshia, aas41@cam.ac.uk

Table I. Dimensions of the mode-localized accelerometer.

Parameter	Value
DETF length	600μm
DETF width	6μm
Lever length	2400μm
Lever width	400μm
Diameter of the coupler	530μm
Proof mass	6.8mg
Area of the proof mass	$73mm^2$
Suspension beam length	700μm
Suspension beam width	5μm
Number of suspension beams	16

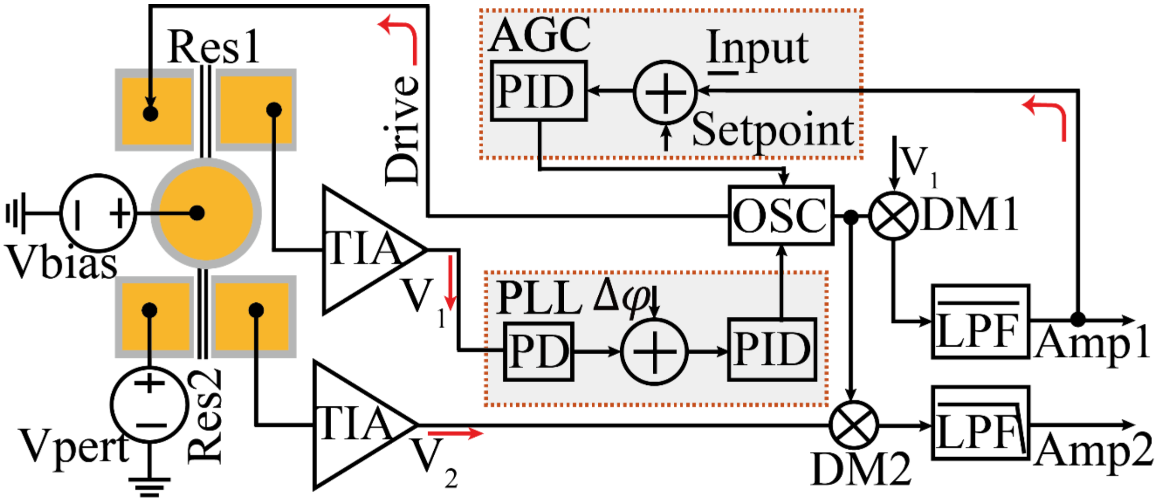

Fig. S1 The electrical test setup. TIA here indicates the transimpedance amplifier, PD the phase detector, PLL the phase-locked-loop, AGC the automatic gain control, OSC the digital oscillator, LPF the low-pass filter, DM the demodulator, and Amp the amplitude.

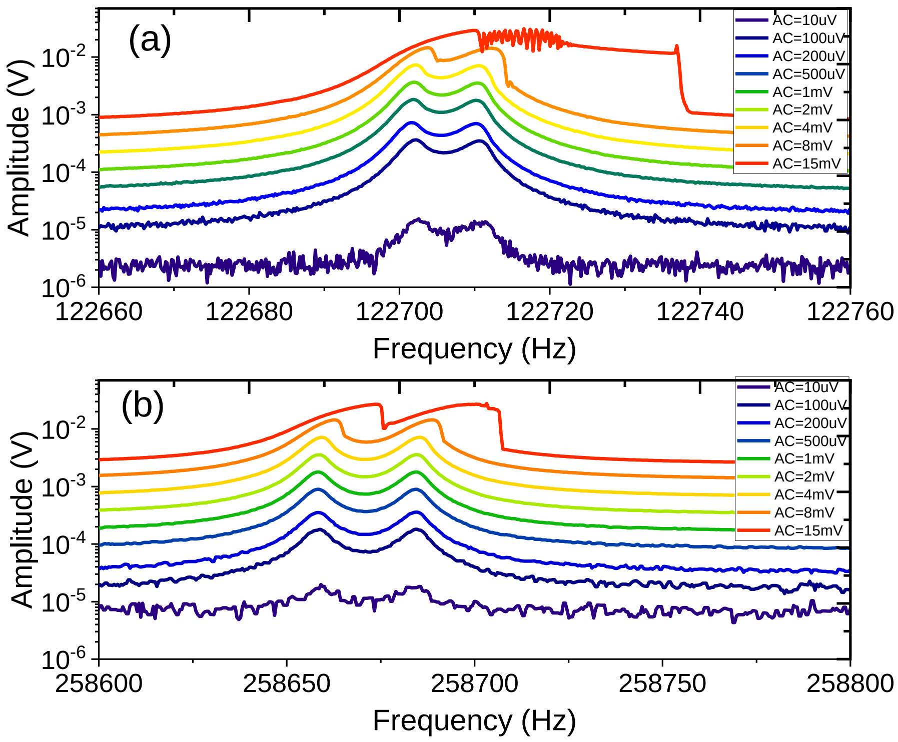

Fig. S2 Frequency responses of Res 2 (the secondary resonator that is not directly driven) at the lower-order (f) and higher-order (g) modes of interests with different drive AC signals, close to the veering point.

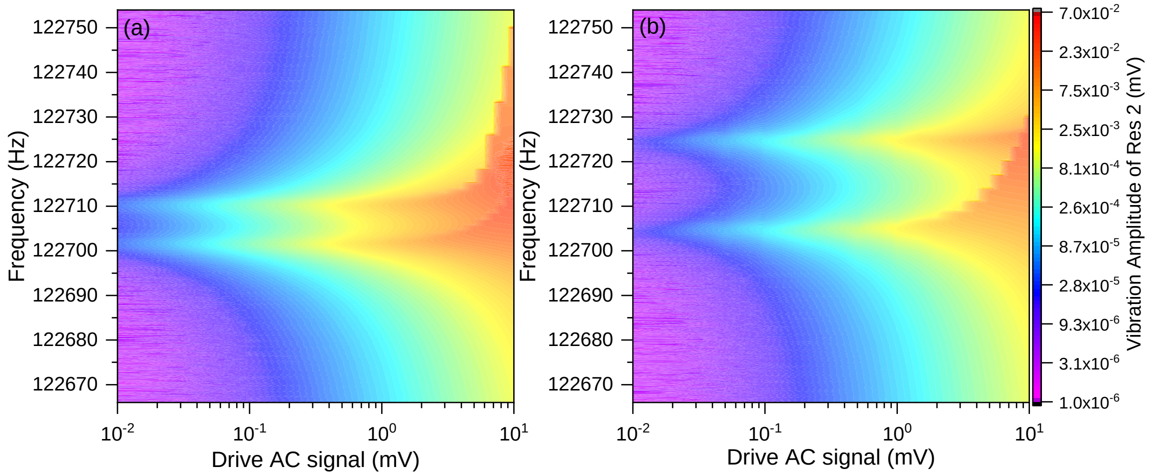

Fig. S3 Experimental measurement of the sweep-up amplitude-frequency responses of Res 2 with different drive AC values, with an initial condition of AR=1.1 (a) and AR=4.2 (b).
